# Evidence for Inbreeding Depression and Pre-Copulatory, but Not Post Copulatory Inbreeding Avoidance in the Cabbage Beetle *Colaphellus bowringi*

**DOI:** 10.1371/journal.pone.0094389

**Published:** 2014-04-09

**Authors:** XingPing Liu, XiaoYun Tu, HaiMin He, Chao Chen, FangSen Xue

**Affiliations:** 1 Institute of Entomology, Jiangxi Agricultural University, Nanchang, Jiangxi Province, China; 2 Life and Sciences College, Jiangxi Normal University, Nanchang, Jiangxi Province, China; Aarhus University, Denmark

## Abstract

Inbreeding is known to have adverse effects on fitness-related traits in a range of insect species. A series of theoretical and experimental studies have suggested that polyandrous insects could avoid the cost of inbreeding via pre-copulatory mate choice and/or post-copulatory mechanisms. We looked for evidence of pre-copulatory inbreeding avoidance using female mate preference trials, in which females were given the choice of mating with either of two males, a sibling and a non-sibling. We also tested for evidence of post-copulatory inbreeding avoidance by conducting double mating experiments, in which four sibling females were mated with two males sequentially, either two siblings, two non-siblings or a sibling and a non-sibling in either order. We identified substantial inbreeding depression: offspring of females mated to full siblings had lower hatching success, slower development time from egg to adult, lower survival of larval and pupal stages, and lower adult body mass than the offspring of females mated to non-sibling males. We also found evidence of pre-copulatory inbreeding avoidance, as females preferred to mate with non-sibling males. However, we did not find any evidence of post-copulatory inbreeding avoidance: egg hatching success of females mating to both sibling and non-sibling males were consistent with sperm being used without bias in relation to mate relatedness. Our results suggest that this cabbage beetle has evolved a pre-copulatory mechanism to avoid matings between close relative, but that polyandry is apparently not an inbreeding avoidance mechanism in *C. bowringi*.

## Introduction

Polyandry is widespread, occurring in many insect species [1], [2]. One of the many proposed explanations for polyandry is that it functions as a mechanism to avoid inbreeding [3], [4], [5], [6]. A growing number of empirical studies have reported that inbreeding may have profound impact on the fitness-related traits of insects [7]. Inbreeding can have strong negative effects on fitness at all life stage, including larval development time [8], [9], survival [10], [11], adult body size and mass [12], [13], [14], adult lifespan [12], [15], fecundity [15], [16], [17], fertility [18], [19], [20], and even immune function [21]. So far, inbreeding depression has been quantified across a range of fitness-related traits in only a limited number of insect species, for example, fruit flies [10], [16], [20], [22], flour beetles [15], [23], seed beetles [8], [11], [17], [24], and field crickets [4], [18], [21], [25], [26]. To better understand the potential impact of inbreeding on mating systems, more comprehensive information on the fitness consequences of inbreeding in other insects is needed.

In some species with limited dispersal of adults from their natal site, females may experience high encounter rates with siblings during mate searching, increasing their risk of inbreeding [27]. Mating with siblings is known to frequently lead to a decline in viability and offspring fitness, known as inbreeding depression, which is caused by increased expression of deleterious recessive alleles and reduced heterozygote advantage [27], [28], [29]. To avoid or reduce the cost of inbreeding, many organisms are expected to have evolved behavioral adaptations preventing or reducing the incidence of mating with their kin [27], [28]. A series of theoretical and experimental studies have demonstrated that individuals recognize and avoid mating with kin via pre-copulatory mate choice [27], [30], [31], [32] and/or females may discriminate against the sperm of kin via post-copulatory mechanisms [25], [26], [33]. However, the costs and benefits of inbreeding depend on the ecological context. Inclusive fitness benefits will favor inbreeding where the kin-selected benefits of devoting reproductive effort to relatives exceed the costs incurred as a result of lower fitness of inbred offspring. Parker [34] (and see [35]) demonstrated that this will occur under even quite high costs of inbreeding and that males require costs of inbreeding to be very high before they will be selected against mating with their siblings. Empirical data from a range of species enable us to examine these predictions.

We have studied inbreeding avoidance and the consequences of inbreeding using the highly promiscuous cabbage beetle, *Colaphellus bowringi* Baly (Coleoptera: Chrysomelidae), as a model. This species is widely distributed in China, where it is as an important pest of Cruciferous vegetables. This beetle is a short-day species undergoing an imaginal summer and winter diapause in the soil [36], [37]. In the field, there are two distinct infestation peaks a year, one in spring with one generation and a second in autumn with three generations [36]. Both sexes copulate on average five times per day [36], [38], [39], [40]. Middle-aged (15-d old) partners have significantly greater mating success in mate choice [41], [42]. Females are very fertile, producing on average around 600 eggs in spring, and 1000 eggs in autumn, with a maximum of 1950 eggs [36]. Therefore, this species provides an excellent model to examine potential pre- and post-copulatory mechanisms of inbreeding avoidance as well as the influence of inbreeding on fitness-related traits.

We first performed mate preference trials to find out whether females can recognize and avoid mating with siblings. Then, we conducted double mating trials in which each female copulated with two non-sibling males, with two sibling males, or a combination of both (following the protocol used by Tregenza & Wedell [4]) to analyze whether there is post-copulatory inbreeding avoidance mechanism in the polyandrous cabbage beetle. Using females and their offspring from these crosses, we analyze whether mating with an inbred male affects mating behaviors, female fecundity and longevity, egg hatching success, the egg-to-adult development time of their offspring, juvenile survival from hatching to adult eclosion and adult body mass.

## Materials and Methods

### Ethics Statement

No specific permits were required for the described field studies. No permission was required to collect insects from China, as *C. bowringi* is an important pest of Cruciferous vegetables in the field, which is neither an endangered nor protected species. Insect culture and observation were conducted in the laboratory of Jiangxi Agricultural University, therefore, no specific permissions were required for this location.

### Beetle Culture and Family Establishment

We used standard cabbage beetle rearing techniques following Xue *et al*. [36]. Naturally diapausing *C. bowringi* adults were collected from vegetable fields in the autumn of 2012 in Xiu-shui county (29°1′N, 114°4′E; about 120 m above sea level), Jiangxi Province, China. They were brought to the laboratory and transferred to large glass bottles (50 cm diameter, 180 cm high) containing soil in which the beetles buried themselves in during dormancy. These glass bottles were kept in laboratory at ambient temperature and humidity. When post-diapause adults emerged from the soil in the spring of 2013, 50 male–female pairs were isolated in individual Petri dishes (height: 1.5 cm, diameter: 9.0 cm) lined with moist filter paper and fresh radish (*Raphanus sativus*) leaves for mating and oviposition. After mating, males were removed from the Petri dishes. Eggs produced by individual females were afterwards transferred into plastic rearing boxes (7.5×7.5×6 cm) containing fresh radish leaves for further development. Consequently, each box represented one full-sib family (F_0_). When first generation adults emerged, males and females of each family were separated and moved into an individual plastic Petri dish. After they reached sexual maturity (5 day after eclosion, [38]), we produced inbred lines by mating a full-sib virgin male and a virgin female from each family. Offspring of these crosses were reared in plastic boxes until adults emerged, and the same procedure was used for two more generations to produce 50 inbred sibling families (*f* = 0.375). We carried out our experiments using the virgin adults of the third inbred generation (F_3_). To obtain virgin males and females, we separated newly emerged F_3_ adults approximately 2 -h from the time of their emergence and kept them in individual plastic Petri dishes. All experiments were conducted in chambers at 25±1°C and L12:D12 photoperiod. Rearing plant was the fresh radish leaves of *R. sativus*. Each experiment began on the fifth day after adult emergence.

### Mate Preference Trials

To determine whether there is a pre-copulatory mechanism of inbreeding avoidance in the polyandrous cabbage beetle, we first performed a female mate preference trial. One day before the trial, males and females were marked with a spot of paint from a silver pen on their right or left elytron, so that their identity could be visually distinguished [41]. At the beginning of the test, two virgin males (a sibling and a non-sibling male) were introduced simultaneously into a Petri dish and permitted a 10 -min acclimatization period. Then a single virgin female was introduced into the Petri dish and allowed to choose between the sibling and non-sibling male. Each female trial ran for 4 -h (from 0800 to 1200 hours) or until she mated. Observations were performed at 5 -min intervals until the copulation ended. When a male successfully copulated, the unpaired male was removed instantaneously and the male with which the experimental female had mated was recorded. The latency before a mating occurred (as the time elapsed from when the beetles were introduced into the Petri dish to when the male inserted his aedeagus into the female’s genitalia) and the copulation duration (the time elapsed from when the male inserted his aedeagus to when he extracted it from female’s genitalia) were recorded. All experimental individuals were used only once.

### Double Mating Trials

To examine potential post-copulatory mechanisms of inbreeding avoidance, we created 50 blocks of four females and four males following the protocol of Tregenza & Wedell [4]. Within a block all four females were full siblings, two males (S1, S2) were full siblings of the four females, and two males (N1, N2) were non-siblings of the four females in the block but were themselves full siblings. Females from each of the 50 blocks were assigned to one of four mating treatments. The first female was mated twice, once to each of her brothers (S1 then S2, thereafter SS). The second female was mated first to one of the non-sibling males and second to one of her brothers (N2 then S1, thereafter NS). The third female was mated first to the other brother and second to the other non-sibling male (S2 then N1, thereafter SN) and the fourth female was mated to full sibling males from a different unrelated family group (N1 then N2, thereafter NN) (see also [18], [19], [25], [43]). Matings were conducted in plastic Petri dishes as described above. Each female trial ran for 4 -h (from 0800 to 1200 hours). After a successful mating, the mated female and male were separated immediately and transferred individually into a new Petri dish lined with moist filter paper and fresh radish leaves. The second round of mating was conducted 24 -h after the first round mating. The first male was then replaced with the second male and the process repeated. Thus, all females received 2 full complements of mating. Following the second mating, the males were removed and females were transferred individually into a new plastic Petri dish lined with moist filter paper and provided with fresh radish leaves for food and oviposition place. Females that failed to mate with the first male they were presented with within the given time were immediately discarded. Similarly, females that mated to the first male, but failed to mate to the second male were also discarded. In this experiment, 22 females (9 from the SS group, 4 from SN group, 6 from NS group and 3 from NN group) from 12 blocks failed to mate with the first and second male that they were presented with. 24 females (9 from the SS group, 6 from SN group, 7 from NS group and 2 from NN group) from 15 blocks died within 10 days after finishing their mating schedule. So the data from 23 remaining blocks were used to further statistical analysis.

### Effect of Mating Treatments on Female Fitness

To estimate the effect of inbreeding on fitness, females from the four treatments (SS, SN, NS and NN) were transferred individually into a new plastic Petri dish after they had finished their mating schedules. Females were monitored daily and their longevity was recorded. Every day for 10 consecutive days, eggs laid by each female in each mating treatment were counted, collected and transferred into a new Petri dish where they were reared under the same conditions as the adults. Incubating eggs were checked daily for hatching and the proportion of hatching eggs was calculated.

### Testing for Viability Benefits of Polyandry through Post-copulatory Female Choice

The rationale for our experimental design follows that of Tregenza and Wedell [4]. The critical prediction is that if polyandry has evolved to reduce viability costs of inbreeding through allowing females to preferentially fertilize their eggs with sperm from more distantly related males then females mating to both related and unrelated males should have eggs and offspring with higher viability than females that do not have the opportunity to exercise post-copulatory choice. Hence the key comparison we are concerned with is between the mean viability of eggs from females in the NN and SS groups compared to the mean for the offspring of females in the SN and NS groups. If egg viability of females in the latter group is higher it is a clear demonstration of a benefit to polyandrous females resulting from costs of inbreeding.

### Effect of Mating Treatments on Offspring Life History Traits

To explore the effects of inbreeding on offspring life history traits, on the fifth day after they had finished their mating schedules, all the eggs from the 23 females in each treatment were combined and 300 were chosen at random and divided into five plastic rearing boxes of 60 eggs each to observe development time and survivorship from egg to adult emergence and adult body mass as indices for offspring performance. Boxes were checked daily and newly emerged females and males of each treatment were weighed on an electronic scale to the nearest 0.01 mg.

### Sperm Precedence

Using the same approach applied for sterile male studies (e.g. [44]), we used the data on hatching rate and egg-to-adult survival to estimate P_2_ values (i.e., the proportion of offspring sired by the second male to mate), from eggs laid by females mated to a sibling and a non-sibling male. We based our estimation on the formula given in Cook *et al*. [41] and Boorman & Parker [45] as follows:
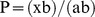
where x is the hatching rate or egg-to-adult survival in the SN treatment or NS treatment, a is the same variable in the NN treatment and b is that variable in the SS treatment. In cases when the mating sequence was SN, the values of P are P_2_ (proportion of offspring sired by the second male), and when the sequence was reversed, the values of P are P_1_ (proportion of offspring sired by the first male, P_1_ = 1–P_2_).

### Statistical Analysis

Data were analyzed using SPSS version 19.0 for Windows (SPSS, Chicago, IL, USA). All percentage data and P_2_ values were normalized prior to statistical analysis by arcsine square root transformation. For the other traits analyses were performed on non-transformed data following checks for normality using Kolmogorov–Smirnov test, and non-parametric statistics were used when normality was violated. A binomial two-tailed test was used to test for female mate preference. A *t*-test was performed to test the differences in mating latency and copulation duration between sibling and non-sibling matings and the differences in the hatching success between NN+SS groups and NS+SN groups. We used the analysis of variance (ANOVA) with Tukey’s HSD post hoc analyses to test the effects of mate relatedness on female fitness (number of eggs, hatching rate and longevity) and offspring fitness-related traits (developmental time, offspring to adult survival and adult body mass). P_2_ data were analyzed using Wilcoxon rank sum test. Data are presented as means ± SE.

## Results

### Female Mate Preference

**Figure 1:**
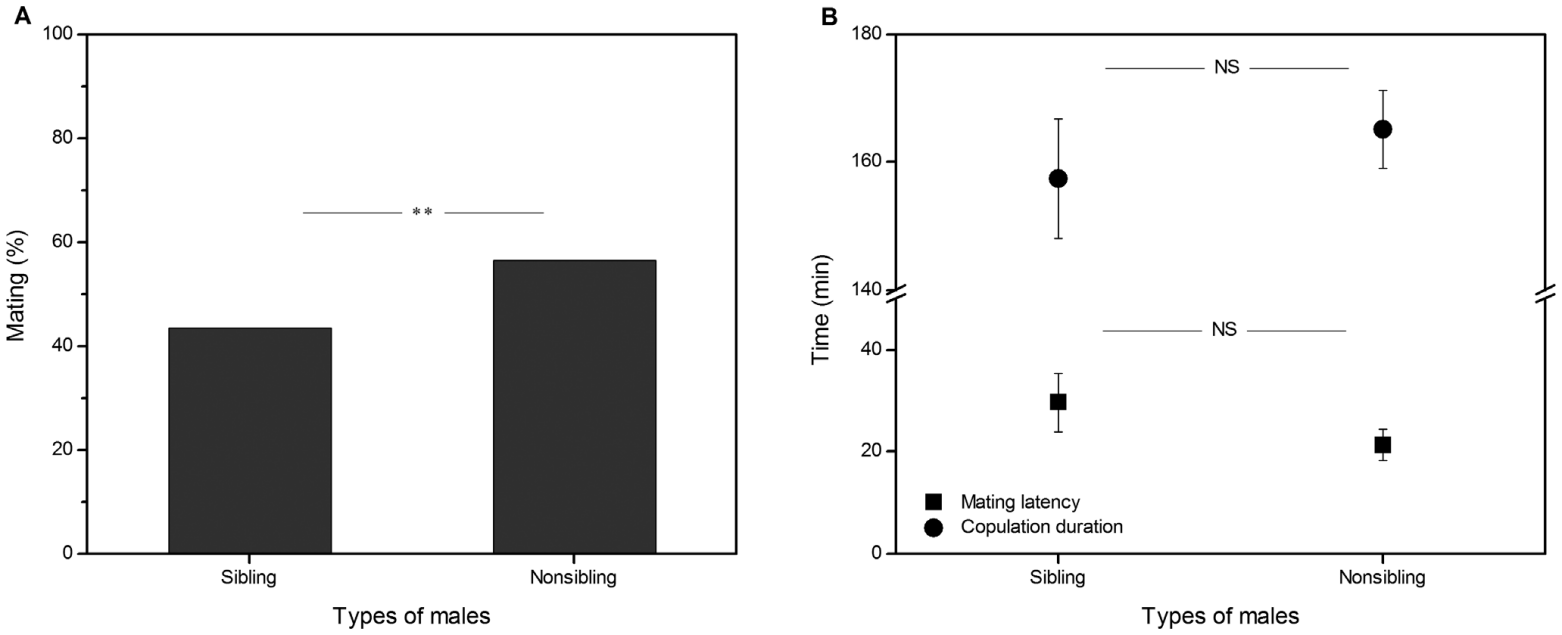
Effect of mate relatedness on female mate preference. Effect of mate relatedness on (A) percentage of mating and (B) mating latency and duration in the cabbage beetle *C. bowringi* in mate preference trials in which a female was simultaneously placed with a sibling and a non-sibling male. Black bars represent percentage mating (mean ± SE, binomial two-tailed test, **: P<0.01). Black circle dots and square dots represent copulation duration and mating latency, respectively (mean ± SE, *t* test, NS: no significant difference).

449 of 542 females mated successfully. When presented simultaneously with a sibling and a non-sibling male, the non-sibling male was significantly more likely to successfully achieve copulation: of the 449 mated females, 195 (43%) mated with sibling males and 254 (57%) mated with non-sibling males (binomial two-tailed test, *P* = 0.006; Fig. 1 A). Mating latency and duration were not different between the sibling (latency = 29.71±1.12 min, duration = 157.37±9.39 min) and non-sibling matings (latency = 21.46±0.82 min, duration = 165.15±6.12 min) (*t*-test, mating latency: *t*_1, 447_ = 0.74, *P* = 0.460; copulation duration: *t*_1, 447_ = 0.58, *P* = 0.503; Fig. 1 B).

### Female Fitness Consequences

**Figure 2:**
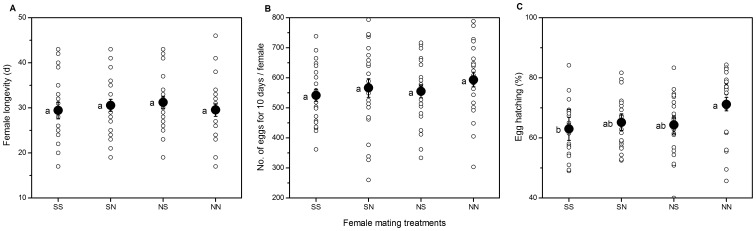
Variation in female fitness parameters among treatment groups. Effect of treatment groups on (A) longevity, (B) fecundity and (C) fertility for doubly mated *C. bowringi* females under four treatments: mated to two sibling males (SS), mated to a sibling followed by a non-sibling male (SN), mated to a non-sibling followed by a sibling male (NS), and mated to two non-sibling males (NN). Black circle dots and error bars represent mean and standard error. Different letters indicate significant differences based on ANOVA with Tukey’s HSD post hoc analyses (*P*<0.05).

In our double mating experiment where females were placed with a single male and given 4 hours in which to mate, there were 13 occasions when a female failed to mate with a sibling male and 9 occasions when she failed to mate with a non-sibling male. Female longevity and the number of eggs produced by females over the 10 days following mating did not differ among mating treatments (ANOVA, longevity: *F*_3, 88_ = 0.34, *P = *0.794; No. of eggs: *F*_3, 88_ = 0.69, *P = *0.559) (Fig. 2 A and 2 B).

### Testing for Viability Benefits of Polyandry through Post-copulatory Female Choice

NN females had the highest hatching success (71.22±2.30%), whereas SS females had the lowest hatching success (63.02±3.81%), SN females and NS females had intermediate hatching success (65.24±2.76%, 64.33±3.04%, respectively). There was a significant difference in egg hatching success among mating treatments (*F*_3, 88_ = 3.71, *P = *0.016; Fig. 2 C). Post-hoc comparisons among mating treatments revealed a significant difference in the hatching success of females mated to two non-sibling males as compared to that of females mated to two sibling males (*P = *0.022). There were no significant differences in egg hatching success between other mating treatments (all *P*>0.05). In relation to our key prediction we found no difference in the mean egg viability of the combined NN and SS families in each block when compared with the mean for the NS and SN groups (*t* test, *t*_44_ = 1.01, *P* = 0.28), indicating that females that had the opportunity to choose between ejaculates from both a sibling and a non-sibling male did not have higher mean egg viability than females that did not have the opportunity to exercise post-copulatory choice in relation to mate relatedness.

### Offspring Life History Traits

**Figure 3:**
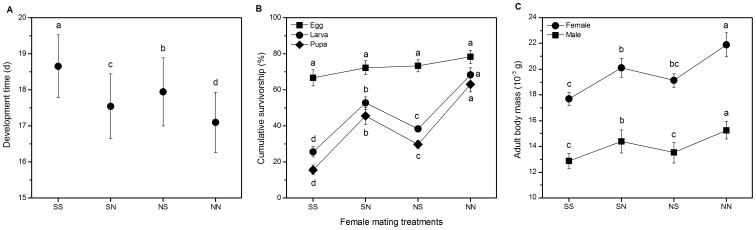
Variation in offspring life history traits among treatment groups. Effect of treatment groups on (A) egg-to-adult developmental time, (B) cumulative survival and (C) adult body mass for offspring from doubly mated *C. bowringi* females under four treatments. Black dots and error bars represent mean and standard error. Different letters indicate significant differences based on ANOVA with Tukey’s HSD post hoc analyses (*P*<0.05).

Female mating treatments significantly affected offspring developmental time (ANOVA, *F*_3, 458_ = 47.33, *P*<0.001; Fig. 3 A). Egg-to-adult development time was shortest in NN offspring, followed by SN, NS and SS offspring (Tukey’s HSD post hoc test, all *P*<0.01). The effects of mating treatments on cumulative survivorship from egg to emergence are shown in Fig. 3 B. At the egg stage, there was no difference in the survival probability of offspring among the four mating treatments (ANOVA, *F*_3, 16_ = 1.64, *P = *0.219). However, female mating treatments significantly affected cumulative survivorship at both larval and pupal stages (larval stage: *F*_3, 16_ = 37.72, *P*<0.001; pupal stage: *F*_3, 16_ = 61.91, *P*<0.001), the cumulative survivorship was highest in NN offspring, followed by SN, NS and SS offspring (Tukey’s HSD post hoc test, larval stage: all *P*<0.05; pupal stage: all *P*<0.01). It is noteworthy that NS offspring had significantly reduced the survival relative to SN offspring at both larval (*P = *0.033) and pupal stages (*P = *0.003). Female mating treatments had a significant effect on offspring body mass of both sexes (female body mass: *F*_3, 219_ = 15.51, *P*<0.001; male body mass: *F*_3, 235_ = 22.25, *P*<0.001; Fig. 3 C), showing that the body mass in NN offspring was significantly greater than those in SN, NS and SS offspring. The body mass of SN and NS offspring showed values intermediate between SS and NN offspring and mating order influenced male body mass (*P = *0.021) but not female body mass (*P = *0.392).

### Sperm Precedence

The pattern of egg hatching success and offspring survival being lower in NS treatments relative to SN treatments is consistent with a situation in which more eggs are fertilized by the 2^nd^ male to mate (when the 2^nd^ male is a sibling eggs have lower hatching rates and offspring survival is reduced). This pattern is strongest in egg-adult survival (Fig. 3 B), allowing us to use these data to estimate mean P_2_ values of 0.63±0.08 across the 5 blocks of SN treatments and 0.71±0.03 in the 5 blocks of NS treatments. These two estimates are independent of one another, but they should be very similar, as they are estimating the same parameter. We confirmed that there was no significant difference between these estimates (Wilcoxon rank sum test W = 7, *P* = 0.289), allowing us to combine them to estimate a mean P_2_ value of 0.67±0.04. We can also attempt a similar analysis using the egg hatching data (Fig. 2 C), although differences among groups relative to variation is much greater in this parameter. Using the egg hatching data, our estimate of P_2_ from the 23 blocks of our NS treatments was 0.59±0.09, and similarly, from the SN treatments our estimate was 0.42±0.09. There was no significant difference between these estimates (Wilcoxon rank sum test W = 205, *P* = 0.174). We can compare the mean P_2_ value from our egg hatching success based analysis of 0.51±0.07 with the 0.67±0.04 estimated from egg-to-adult survival group, revealing no significant difference between the two estimates (Wilcoxon rank sum test W = 252, *P* = 0.638).

## Discussion

We demonstrate substantial fitness costs of inbreeding across the life-history of *C. bowringi* with the potential to drive the evolution inbreeding avoidance mechanisms. The overall pattern of offspring fitness traits with values associated with the highest fitness in female mating to two non-sibling males (NN) and lowest values when mated to two full sibling males (SS) is consistent across traits. Firstly, the estimated mean hatching success of 63% for SS females was significantly less than the 71% of NN females (Fig. 2 C). Secondly, SS offspring exhibited a significant delay in development time from egg to adult emergence and a significant reduction in cumulative survivorship in both larval and pupal stages (Fig. 3 A and B). Thirdly, the body mass of both sexes in SS offspring was significantly smaller than those in NN (Fig. 3 C).

There are widespread theoretical predictions and growing empirical evidence that insects have evolved mechanisms to avoid breeding with close kin [7], [20], [27], [33]. Pre-copulatory kin recognition has been reported for several insect and mite species. For example, in the gregarious cockroaches *Blattella germanica* [30], the yellow mealworm beetle, *Tenebrio molitor* [32], field cricket *Gryllus bimaculatus* [46] and two-spotted spider mite *Tetranychus urticae* [47], when presented simultaneously with a sibling and a non-sibling male, females mated significantly more often with the non-sibling male. However, a lack of pre-copulatory inbreeding avoidance has been also found in the Glanville fritillary butterfly *Melitaea cinxia* [48], the bruchid beetle Callosobruchus maculates [*49*] and the parasitoid wasp *Cotesia glomerata* [50]. Our mate preference experiment showed that females mated significantly more often with non-sibling males than sibling males (Fig. 1 A). Because our design means that there are no systematic differences between males in the two treatment groups other than whether or not they are related to the female, we can rule out male-male competition as an explanation for this result. It is possible that males are less willing to mate with their siblings than with an unrelated female so we cannot exclude male mate choice (or mutual mate choice) as an alternative explanation, although the much greater investment by females than males in reproduction suggests that female mate choice is much more likely than male mate choice as demonstrated by Parker [34] with specific reference to inbreeding avoidance.

It is not known how common interactions between full siblings during mate searching are in natural populations of this beetle. Individuals are highly mobile [38] which will reduce encounters between full siblings although matings among relatives have been shown to occur in natural populations of highly dispersive insects [51]. Other factors may promote interactions between full siblings: Firstly, the large numbers of eggs produced by a single female certainly create potentially large numbers of siblings. Secondly, the chances of a female encountering a sibling will be proportional to the number of males they can potentially mate with, which in this species is very high: females can mate with as many as 5 males per day and an average of 40 times during the first 10 days of mating [36], [38]. Thirdly, the species has a complex and highly variable pattern of diapause, with 2 separate diapause periods, which can vary in length from a few months to several years [36], [52]. If the tendency to enter and leave diapause is heritable this will tend to increase the chances of siblings encountering one another, and similarly, if diapause patterns are very sensitive to the timing of hatching then cohorts of siblings may be more likely to encounter one another as adults.

Female *C. bowringi* do not appear to use post-copulatory mechanisms to bias sperm usage towards non-sibling males. Offspring of females mated to two siblings had lower hatching success than those of females mated to two non-sibling males, indicating the existence of inbreeding depression. However, we found no difference in hatching success between offspring of females mated to a sibling and a non-sibling male in either order and those of females mated to two siblings. Furthermore, if females can bias paternity in favor of sibling or non-sibling males, we would expect to see a significant difference in P_2_ between females mated to a sibling male first and a sibling male second (SN and NS). However, in our sperm precedence analysis, we did not find any differences in P_2_ between these groups using our data on egg-to-adult survival or our data on egg hatching rate. The lack of post-copulatory inbreeding avoidance we observe has been also found in the black field cricket, *Teleogryllus commodus* [18] and the fruit fly *Drosophila melanogaster* [20]. It is tempting to speculate that the existence of pre-copulatory inbreeding avoidance may be related to the lack of post-copulatory inbreeding avoidance, on the basis that a strong pre-copulatory barrier to inbreeding will reduce selection in favor of subsequent potential avoidance adaptations. However, it is clear that pre-copulatory inbreeding avoidance is far from complete, so this is unlikely to represent a complete explanation. A greater body of literature on this subject will allow future meta-analytical studies to examine the possibility of a general pattern of a negative relationship between pre- and post-copulatory avoidance mechanisms.

We found no evidence that *C. bowringi* females show any differences in longevity or fecundity when mated with siblings relative to non-siblings (Fig. 2 A and B). *C. bowringi* females typically mate with more than 2 males during their lifetime under semi-natural and laboratory conditions [36], [38], whether two matings is enough to cause changes in female longevity between sibling and non-sibling males is unclear. According to our observations, an unmated *C. bowringi* female can produce almost the same number of eggs as a mated female [40].

Finally we demonstrate that in species with strong and quantifiable inbreeding depression, it is possible to estimate the mean paternity success of the 2^nd^ male to mate (P_2_) using inbreeding depression as a marker of paternity, which provides a novel and potentially widely applicable approach to estimate this parameter without using genetic markers or sterilizing individuals.

In conclusion, our study provides evidence for pre-copulatory inbreeding avoidance mechanisms by *C. bowringi* females, as females mated significantly more often with non-sibling males than sibling males. Although we identified substantial inbreeding depression in this polyandrous cabbage beetle, we did not find any evidence of post-copulatory inbreeding avoidance mechanisms, as males contributed equally to fecundation irrespective to their relatedness with the female through our sperm precedence analysis. Thus, our data suggests that polyandry is not the main mechanism to avoid inbreeding in this beetle species.
