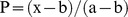# Correction: Evidence for Inbreeding Depression and Pre-Copulatory, but Not Post Copulatory Inbreeding Avoidance in the Cabbage Beetle *Colaphellus bowringi*

**DOI:** 10.1371/journal.pone.0103052

**Published:** 2014-07-14

**Authors:** The PLOS ONE Staff

The formula in the section “Sperm Precedence” in the Materials and Methods is incorrectly displayed. The correct formula is shown below.